# Artificial gauge fields in the *t*-*z* mapping for optical pulses: Spatiotemporal wave packet control and quantum Hall physics

**DOI:** 10.1126/sciadv.adj0360

**Published:** 2023-10-20

**Authors:** Christopher Oliver, Sebabrata Mukherjee, Mikael C. Rechstman, Iacopo Carusotto, Hannah M. Price

**Affiliations:** ^1^ School of Physics and Astronomy, University of Birmingham, Edgbaston, Birmingham B15 2TT, UK.; ^2^ Department of Physics, Indian Institute of Science, Bangalore 560012, India.; ^3^ Department of Physics, The Pennsylvania State University, University Park, PA 16802, USA.; ^4^ Pitaevskii BEC Center, INO-CNR and Dipartimento di Fisica, Università di Trento, I-38123 Trento, Italy.

## Abstract

We extend the *t-z* mapping of time-dependent paraxial optics by engineering a synthetic magnetic vector potential, leading to a nontrivial band topology. We consider an inhomogeneous 1D array of coupled optical waveguides and show that the wave equation describing paraxial propagation of optical pulses can be recast as a Schrödinger equation, including a synthetic magnetic field whose strength can be controlled via the spatial gradient of the waveguide properties across the array. We use an experimentally motivated model of a laser-written array to demonstrate that this synthetic magnetic field can be engineered in realistic setups and can produce interesting physics such as cyclotron motion, a controllable Hall drift of the pulse in space or time, and propagation in chiral edge states. These results substantially extend the physics that can be explored within propagating geometries and pave the way for higher-dimensional topological physics and strongly correlated fluids of light.

## Teaser

Classical optics can mimic the quantum motion of particles in a magnetic field, with consequences for controlling light.

## INTRODUCTION

A remarkable result of paraxial optics is that the electromagnetic field of paraxially propagating classical light can be described by a Schrödinger-like equation (*1*). In this equation, the usual time evolution of a wave function is replaced by the propagation of the electric field envelope along the optical axis, *z*, of the medium. In practice, a major platform for investigating paraxial propagation is arrays of coupled optical waveguides, laser-written into a substrate (*2*). In general, these propagating geometries can be used to emulate a variety of single-particle quantum phenomena (*3*–*8*) or mean-field interacting physics if the medium is nonlinear (*9*–*15*). This interacting physics includes Bose-Einstein condensates of photons and opens the way to studies of fluids of light (*16*–*18*), with interesting perspectives toward quantum features (*19*).

One exciting avenue that has been explored intensively over the past 15 years is that of topological photonics (*20*, *21*). In this field, the physics of topological phases of matter, originally discovered within the context of electrons in solids, is applied to photonics. Propagating geometries have proven to be a very fruitful platform in this context, with a major early achievement (*22*) being the investigation of Floquet topological insulators in which a honeycomb array of waveguides acquires a nontrivial topology when a helical patterning of the waveguides is introduced. Because the propagation distance *z* plays the role of the temporal evolution, the breaking of the translational symmetry along the *z* direction of the helical waveguide system is analogous to the breaking of time-translation symmetry in a two-dimensional (2D) tight-binding model of electrons under a temporally periodic modulation (*23*). More generally, similar propagating geometries have proven to be a powerful tool for studying a wide variety of topological physics, including the investigation of the interplay between nonlinearity and topology (*24*–*26*), topological physics in non-2D geometries (*27*–*30*), non-Hermitian effects in topology (*31*, *32*), disorder (*33*, *34*), Thouless pumping schemes (*35*, *36*), transport (*37*), and quantum walks (*38*).

So far, most, if not all, works on topological photonic effects using propagating geometries have used monochromatic light and so do not make substantial use of the temporal dynamics associated with an optical pulse. From paraxial optics (*1*), it is well known that the Schrödinger-like equation describing the propagation of an optical pulse in a dispersive medium also includes a second-order time derivative term, with the same structure as a kinetic energy term in quantum mechanics. This allows us to interpret time *t* as an additional spatial dimension in addition to the transverse *x*, *y* ones, while the coordinate *z* along the propagation direction plays the role of a time. This interchange of the role of space and time is known as the *t-z* mapping and has also been investigated at the quantum level in (*19*, *39*–*41*).

In this work, we propose a novel configuration based on an array of coupled optical waveguides where a synthetic gauge field naturally appears when the temporal dynamics of an optical pulse is taken into account under the *t-z* mapping. In particular, we consider propagation across a 1D array of coupled optical waveguides with slightly different properties and show that the 2D wave equation for the classical optical field propagation in a mixed spatial-*j*/temporal-*t* plane has the form of a Schrödinger equation including a synthetic vector potential term as in the coupled wire model of quantum Hall physics (*42*–*44*). A realistic configuration resulting in a sizable synthetic magnetic field and a nontrivial band topology is put forward, and experimentally accessible signatures of the magnetic effects are pointed out. These include a cyclotron motion of light wave packets in the spatiotemporal *j-t* plane, a Hall drift in response to additional synthetic electric fields in either the spatial or the temporal direction, and unidirectional propagation in chiral edge states. As compared to previous schemes (*20*, *45*) for synthetic magnetic fields and synthetic dimensions in arrays of microcavities (*46*, *47*) or waveguides (*48*–*51*), our proposal has the crucial advantage that it does not require a dynamical modulation of the system and provides a new tool for the manipulation of the spatiotemporal shape of optical wave packets. Moreover, the local interactions in our system suggest exciting prospects for strongly correlated fluids of photons if the interaction strength can be scaled up.

The structure of the article is the following: We first summarize the mapping of the paraxial wave equation onto the Schrödinger equation with a synthetic magnetic field. The quantum Hall coupled wire model is then reviewed, and an experimentally realistic configuration for realizing it is presented. Observable signatures of the synthetic magnetic field and the nontrivial band topology are next presented. We finish by sketching conclusions and perspectives toward quantum topological photonics and quantum fluids of light.

## RESULTS

### Mapping the paraxial wave equation onto a Schrödinger equation with a synthetic magnetic field

In this first section, we review the derivation of the well-known wave equation for the paraxial propagation of a pulse though an array of coupled waveguides (*1*). For suitably designed inhomogeneous arrays, we then map the wave equation onto a Schrödinger equation for a particle in a synthetic magnetic field, where time plays the role of a synthetic spatial dimension and propagation through the array corresponds to time evolution. This equation will be our workhorse for the rest of the paper.

**Fig. 1.**
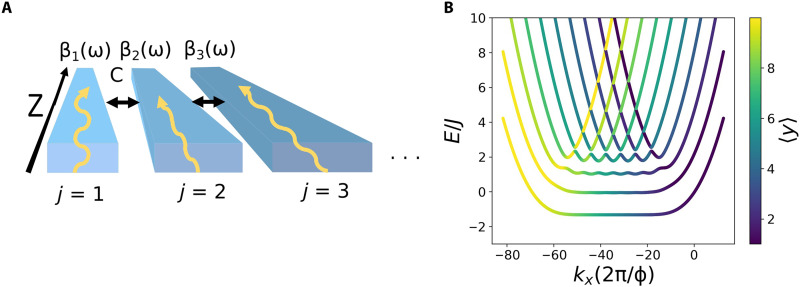
Our basic setup and target quantum Hall model. (**A**) A schematic of our proposed setup consisting of an inhomogeneous 1D array of coupled single-mode waveguides. Each waveguide has a propagation constant β_*j*=1,…,*N*_(ω), and neighboring waveguides are coupled together evanescently with a coupling strength *C*. The waveguide axis lies along the *z* direction. The waveguide properties (e.g., the width and refractive index) vary across the array to engineer a nontrivial synthetic magnetic field. (**B**) Example of the band structure of a quantum Hall coupled wire model formed by *N* = 10 wires with periodic boundary conditions in the continuous *x* direction. For each state of the band structure, the color coding indicates its average spatial position along *y*. We measure *k_x_* in units of 1/*l_B_*, where $l_{B}=\sqrt{\hbar /qB}$ is the magnetic length. The one free parameter of the Hamiltonian, the ratio of the two energy scales *r* ≡ (ℏ^2^/$ml_{B}^{2}$)/(ℏ*J*), is set to unity. We find, at low energies, dispersionless bulk Landau levels and chiral states localized on the system edge in the gaps, as is characteristic of a quantum Hall model.

Consider an optical pulse propagating through a 1D array of *j* = 1, …, *N* single-mode waveguides whose optical axis points in the *z* direction, as shown schematically in Fig. 1A. We can write the electric field in waveguide *j* as

$$E_{j}(r,t)=e_{j}(x,y)a_{j}(z,t)e^{i[\beta_{j_{\mathrm{ref}}}(\omega_{0})z-\omega_{0}t]}$$ (1)

where *e_j_*(*x*, *y*) is the electric field profile in the plane perpendicular to the optical axis, ω_0_ is the pulse carrier frequency, and β_*j*_ref__(ω_0_) is the carrier propagation constant in the *j* = *j*_ref_ waveguide used as a reference. We choose this decomposition to separate out the envelope *a_j_*(*z*, *t*), which, within the paraxial approximation, is assumed to be slowly varying as a function of *z* and *t*. We do not consider nontrivial polarization effects, so *E_j_* can represent any polarization component of the electric field. We assume that the waveguides are effectively single mode, meaning that any excited modes are well separated from the fundamental mode in propagation constant so that they play no role in the dynamics. We also neglect loss and disorder in the optical medium.

In frequency space, the propagation of the pulse is described by the coupled equations for the field amplitudes in the different waveguides

$$i\frac{\partial {\tilde{a}}_{j}}{\partial z}=-[\beta_{j}(\omega^{\prime }+\omega_{0})-\beta_{j_{\mathrm{ref}}}(\omega_{0})]{\tilde{a}}_{j}-C({\tilde{a}}_{j-1}+{\tilde{a}}_{j+1})$$ (2)

where ${\tilde{a}}_{j}(z,\omega^{\prime })$ is the Fourier transform of *a_j_*(*z*, *t*) with respect to *t* in terms of the frequency variable ω′ = ω − ω_0_ and *C* is the evanescent coupling between neighboring waveguides. For simplicity, this coupling is assumed to be frequency independent in the range of interest and constant across the array. In typical implementations where the coupling strength is controlled by the spacing between waveguides, this latter condition may require an appropriate modulation of the spacing across the array to compensate for the variable size of the waveguides.

We now Taylor-expand β*_j_*(ω) about ω_0_

$$\beta_{j}(\omega )\approx \beta_{j}(\omega_{0})+\frac{d\beta_{j}(\omega )}{d\omega }{{|}_{\omega_{0}}(\omega -\omega_{0})+\frac{1}{2}\frac{d^{2}\beta_{j}(\omega )}{d\omega^{2}}|}_{\omega_{0}}{\left(\omega -\omega_{0}\right)}^{2}$$ (3)

where we neglect terms O[(ω − ω_0_)^3^] and higher. We then identify

$${v_{g}^{\left(j\right)}={\left[\frac{d\beta_{j}}{d\omega }{|}_{\omega_{0}}\right]}^{-1},D_{j}=\frac{d^{2}\beta_{j}}{d\omega^{2}}|}_{\omega_{0}}$$ (4)

as the group velocity and the group velocity dispersion in waveguide *j*, respectively. Substituting this expansion into Eq. 2 and Fourier-transforming back to the time domain produces the wave equation

$$i\frac{\partial a_{j}}{\partial z}=\frac{D_{j}}{2}\frac{\partial^{2}a_{j}}{\partial t^{2}}-\frac{i}{v_{g}^{\left(j\right)}}\frac{\partial a_{j}}{\partial t}-[\beta_{j}(\omega_{0})-\beta_{j_{\mathrm{ref}}}(\omega_{0})]\;a_{j}-C(a_{j+1}+a_{j-1})$$ (5)

which can be easily mapped onto a Schrödinger equation.

To this purpose, we transform to a frame comoving with the group velocity $v_{g}^{\left(\mathrm{ref}\right)}$ ≡ *d*β_*j*_ref__/*d*ω∣_ω_0__ in the *j*_ref_ reference waveguide using a Galilean transformation with space and time interchanged, ζ ≡ *z* and τ ≡ *t* − *z*/$v_{g}^{\left(\mathrm{ref}\right)}$. After completing the square to eliminate the first time derivative, Eq. 5 becomes

$$i\frac{\partial a_{j}^{\prime }}{\partial \zeta }=\frac{1}{2m_{j}}{\left[-i\frac{\partial }{\partial \tau }-A_{j}^{\left(\tau \right)}\right]}^{2}a_{j}^{\prime }+V_{j}\;a_{j}^{\prime }-C(a_{j+1}^{\prime }+a_{j-1}^{\prime })$$ (6)

where we have defined

$$m_{j}=-\frac{1}{D_{j}},A_{j}^{\left(\tau \right)}=\frac{1}{D_{j}}\left[\frac{1}{v_{g}^{\left(j\right)}}-\frac{1}{v_{g}^{\left(\mathrm{ref}\right)}}\right],V_{j}=\frac{1}{2D_{j}}{\left[\frac{1}{v_{g}^{\left(j\right)}}-\frac{1}{v_{g}^{\left(\mathrm{ref}\right)}}\right]}^{2}-[\beta_{j}(\omega_{0})-\beta_{j_{\mathrm{ref}}}(\omega_{0})]$$ (7)

and where $a_{j}^{\prime }$(ζ, τ) is the electric field envelope in the comoving frame (denoted from now on by the prime symbol).

This set of equations has the form of coupled Schrödinger equations, where propagation along the optical axis of the waveguide array plays the role of time evolution, as we expect from the *t-z* mapping. The particle with unit charge moves in a 2D system with one discrete (*j*) dimension and one continuous (τ) dimension: In the former direction, the hopping amplitude is *C*; in the latter, the particle has a (position-dependent) mass *m_j_* determined by the group velocity dispersion of the waveguides.

On top of this, the particle experiences a magnetic vector potential in the τ-direction $A_{j}^{\left(\tau \right)}$ and a scalar potential *V_j_*. Crucially, the magnetic vector potential is oriented along τ and is waveguide dependent, so nontrivial magnetic field effects can be engineered by introducing a spatial gradient of the waveguides’ characteristics across the array. Because time reversal is automatically broken by propagation, our proposal does not require any dynamical modulation of the system (*52*, *53*). In contrast to models where a synthetic dimension is encoded in momentum-space quantities (*52*, *53*) or in the light frequency (*46*–*51*), our proposed topological model is based on propagation in real space-time coordinates, which is of utmost interest in the long term to integrate local nonlinearities and realize strongly interacting photon models.

We also note that if one is not to make the Taylor expansion in Eq. 3 and wishes to keep the complete form of the waveguide dispersion β*_j_*(ω), then one obtains in the comoving frame the following form of the evolution equation in Fourier space

$$i\frac{\partial {\tilde{a}}_{j}^{\prime }}{\partial \zeta }=-\left[\beta_{j}(\omega^{\prime }+\omega_{0})-\beta_{j_{\mathrm{ref}}}(\omega_{0})-\frac{\omega^{\prime }}{v_{g}^{\left(\mathrm{ref}\right)}}\right]\;{\tilde{a}}_{j}^{\prime }-C({\tilde{a}}_{j+1}^{\prime }+{\tilde{a}}_{j-1}^{\prime })$$ (8)

For both this and our Schrödinger equation (Eq. 6), the propagation eigenmodes of the array are then obtained by searching for stationary solutions of this equation in the form

$${\tilde{a}}_{j}^{\prime }(\zeta ,\omega^{\prime })=e^{i\;\text{Δ}\beta^{\prime }\;\zeta }\;{\tilde{a}}_{j}^{\prime }(\omega^{\prime })$$ (9)

where the propagation constant in the comoving frame, Δβ′, is related to the laboratory frame one by

$$\beta (\omega )=\beta_{j_{\mathrm{ref}}}(\omega_{0})+\frac{\omega -\omega_{0}}{v_{g}^{\left(\mathrm{ref}\right)}}+\text{Δ}\beta^{\prime }(\omega )$$ (10)

For visualization purposes, in the following, we will study the dispersion in terms of Δβ′, where Δ highlights that we consider propagation constants relative to a reference.

Last, we note that, in the simplest limit where the mass is constant across the array (*m_j_* = *m*), the scalar potential vanishes (*V_j_* = 0), and the vector potential displays a linear gradient along *j*, $A_{j}^{\left(\tau \right)}$ = −*ℬj*, with ℬ being a uniform magnetic field, this equation reduces to the well-known quantum Hall coupled wire model. In the next section, we briefly move away from optics to review the physics of this model in general. This simple model will serve as an intuitive guide for the following developments of the paper.

### The quantum Hall coupled wire model

In the quantum Hall coupled wire model, a charged particle is subject to a uniform magnetic field and moves within a system of *N* coupled wires: The particle can either freely move along each wire (as denoted by the continuous dimension, *x*) or hop between neighboring wires (along the discrete dimension, *y*) (*42*). Hence, this model lies between the fully continuous Landau levels for a particle in free space and the fully discrete Harper-Hofstadter model for a particle on a 2D square lattice (*54*). Originally, the coupled wire model was investigated in the context of the fractional quantum Hall effect (*42*–*44*); its interest is related to the ability to control the band flatness by varying the hopping between wires. Recently, it has also been realized experimentally using the internal atomic states as a (discrete) synthetic dimension in addition to a real spatial dimension (*55*).

In mathematical terms, the coupled wire model is summarized by the Hamiltonian

$$\hat{H}=\frac{\hbar^{2}}{2m}{\left({\hat{k}}_{x}+\frac{qB}{\hbar }y\right)}^{2}+\hbar J\sum_{y}(|x,y+a\rangle \langle x,y|+H.C.)$$ (11)

where *q* and *m* are the particle’s charge and mass along *x*, respectively, and *J* is the hopping between adjacent sites along the discrete dimension *y*, of lattice spacing *a*. The magnetic field is uniform and equal to *B*, and a Landau gauge is adopted with the vector potential oriented along the continuous *x* direction, $A=-By{\hat{e}}_{x}$. In our *t-z*–mapped Schrödinger equation (Eq. 6), the waveguide index *j* corresponds to *y* and the time in the comoving frame τ corresponds to *x*. The hopping *J* corresponds to the evanescent coupling *C* between neighboring waveguides, and the particle mass corresponds to the (reciprocal of the) group velocity dispersion. The magnetic vector potential −*By* corresponds to our $A_{j}^{\left(\tau \right)}$. However, we emphasize that, as highlighted above, the quantum Hall coupled wire model is a general model that is of interest to several communities. We also note that, if we measure *k_x_* in units of the inverse of the magnetic length $l_{B}\equiv \sqrt{\hbar /qB}$ and the Hamiltonian in units of ℏ*J*, there is only one free parameter, namely, the ratio of the two kinetic energy scales *r* ≡ (ℏ^2^/$ml_{B}^{2}$)/(ℏ*J*).

To gain some intuition for the physics of the coupled wire model, we take periodic boundary conditions along the continuous (*x*) direction, as in (*42*). This allows us to exploit the conserved momentum *k_x_* to diagonalize the Hamiltonian. An example of the coupled wire model band structure calculated from the above procedure is shown in Fig. 1B, where the coloring of the states denotes their average position with respect to the discrete dimension.

Physically, the most important features of this band structure are the existence, at low energies, of dispersionless bulk band states and of unidirectionally propagating edge states. The former are localized in the discrete bulk (green/blue coloring) and have an almost constant energy, corresponding to no group velocity; hence, they are a semidiscrete analog of the flat Landau levels of charged particles subject to homogeneous magnetic field in free space. The latter are localized on the edges of the system (yellow/purple coloring), and their *k_x_*-dependent energy falls in the gaps between the flat levels; these states are one-way chiral edge states with nonzero group velocity and are protected by the nontrivial topology of the model, i.e., the nonzero Chern number of the bands.

Intuitively, the appearance of these two types of states can be simply understood from Eq. 11. In the absence of the interwire coupling *J*, the dispersion consists of *N* parabolae (corresponding to each of the *N* wires), which are equally spaced along *k_x_* because of the uniform magnetic field, and their minima have all the same energy. As energy increases, each parabola crosses sequentially with those of the neighboring wires, except for the ones at the edges of the array, where neighbors are only present on one side. Turning on the interwire coupling *J* then lifts the degeneracies around the crossings, mixing states and giving rise to the flat bulk bands in the center of the band structure and the localized one-way states at the edges that are visible in Fig. 1B.

Having reviewed the physics of this general coupled wire model, we return to optics in the next section, where we will see how the model can be naturally realized by the coupled Schrödinger equations (Eq. 6) in a suitably designed waveguide array. We will also assess the impact of additional features such as the on-site potential *V_j_* and position-dependent mass *m_j_* terms.

### An experimentally motivated model of a laser-written waveguide array

From our discussion in the previous sections, the key ingredient to generate the synthetic magnetic field for photons is to design the *j* dependence of the waveguide dispersion β*_j_*(ω) to obtain a finite gradient along *j* of the group velocity. To this purpose, we consider a model of *N* waveguides embedded in a medium of frequency-dependent refractive index *n*_0_(ω), and for simplicity, we restrict our attention to a single transverse coordinate *x*. Within each waveguide *j*, light is confined by a (frequency-independent, for simplicity) lateral spatial profile of the refractive index. More precisely,

$$n_{j}(x,\omega )=n_{0}(\omega )+\delta n_{j}\;\exp\left[-{\left(\frac{x^{2}}{2\sigma_{j}^{2}}\right)}^{m}\right]$$ (12)

where for concreteness and with no loss of generality, we consider the specific example of the refractive index *n*_0_(ω) of fused silica glass (*56*) used in many recent experiments (*57*). Experimentally motivated *m* = 10 and δ*n_j_* > 0 values are taken for the super-Gaussian power of the spatial profile and the refractive index shift, respectively.

To obtain the synthetic magnetic field, different values of the width σ*_j_* and the refractive index depth *n_j_* ≪ 1 must be taken for the different waveguides. In experiments, these parameters are controlled by varying the speed at which the optical medium is translated across the beam used for writing.

We calculate the dispersions β*_j_*(ω) for our refractive index profile by numerically solving the Helmholtz equation for our refractive index profile (*1*, *20*)

$$i\frac{\partial {\tilde{a}}_{j}(x,z,\omega )}{\partial z}=-\frac{c}{2n_{0}(\omega )\omega }\frac{\partial^{2}{\tilde{a}}_{j}(x,z,\omega )}{\partial x^{2}}-\frac{\omega }{c}\delta n_{j}\;\exp\left[-{\left(\frac{x^{2}}{2\sigma_{j}^{2}}\right)}^{m}\right]{\tilde{a}}_{j}(x,z,\omega )$$ (13)

which has the form of a Schrödinger equation in which the refractive index perturbation plays the role of a potential well. We write ${\tilde{a}}_{j}(x,z,\omega )={\tilde{a}}_{j}(x,\omega )e^{i\delta \beta_{j}(\omega )z}$, where δβ*_j_*(ω) is the part of the propagation constant due to the waveguide itself, and we diagonalize the resulting equation. We choose the fundamental mode and verify that the other modes are well separated in propagation constant. This produces the total dispersion β*_j_*(ω) ≡ *n*_0_(ω)ω/*c* + δβ*_j_*(ω). We can then use our mapping (Eq. 10) to change to the comoving frame.

**Fig. 2.**
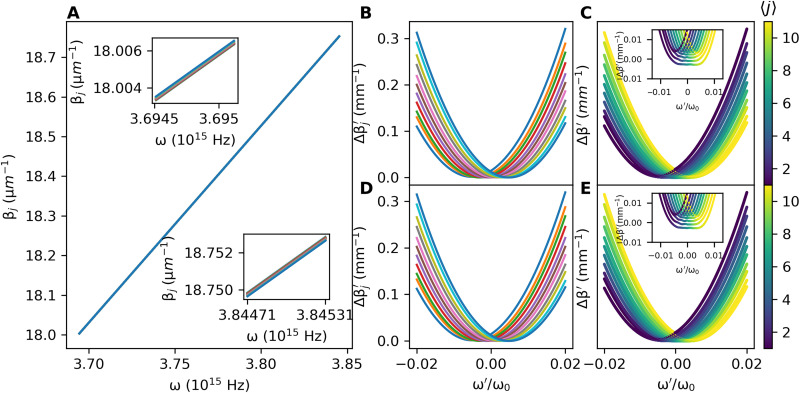
Propagation constants for the model. (**A**) Propagation constants in the laboratory frame for an array of *N* = 11 uncoupled waveguides with refractive index profile as in Eq. 12, after the waveguide widths and refractive index perturbations have been tuned to make the comoving frame coupled array band structure have similar features as the quantum Hall coupled wire model. The insets show two different frequency ranges, with the order of the curves reversed between the two, showing that the curves intersect each other. (**B**) The uncoupled propagation constants in (A) transformed into the comoving frame as described in the text. We find minima at approximately the same propagation constant value and approximately uniform spacing in frequency. Physically, these correspond to having an almost-constant scalar potential and a near-uniform magnetic field, respectively. (**C**) The dispersion curves in (B) when a nearest-neighbor evanescent coupling of *C* = −0.002 mm^−1^ is included, showing avoided crossings. The color of each state denotes the expectation value of its position with respect to the discrete direction. We see that this band structure includes bulk states (green) that are nearly dispersionless (see inset) and chiral edge modes (purple/yellow) within the gap like in the coupled wire model. (**D** and **E**) The uncoupled (D) and coupled (E) comoving frame dispersions calculated from our Schrödinger equation (Eq. 6), showing excellent agreement with (B) and (C). Throughout this figure, the parameters are as in Fig. 5.

We then need to adjust our free parameters of the array, δ*n_j_* and σ*_j_*, to make our comoving frame dispersion curves as close as possible to the coupled wire model, i.e., a uniform horizontal spacing between the curves corresponding to a uniform magnetic field, and the minima of the curves all being level vertically, corresponding to no on-site potential. The method for doing this is described in Materials and Methods. The end results in the laboratory frame are plotted in Fig. 2A and in the comoving frame in Fig. 2B.

Including the evanescent coupling *C* into our uncoupled dispersion and diagonalization of the comoving frame equation (Eq. 8) gives the eigenmodes shown in Fig. 2C and, in a magnified view, in the inset of this panel. The qualitative resemblance with the quantum Hall coupled wire model is apparent: The bottom of the dispersion forms isolated bands corresponding to almost flat Landau levels that transform into edge states on the sides of the dispersion. The color scale highlights the spatial location of the different states: As expected, Landau levels are localized in the bulk, while the chiral edge states sit on the extreme waveguides *j* = 1 (purple) and *j* = *N* (yellow).

For comparison, we also calculate the band structure for the waveguide array using our Schrödinger equation (Eq. 6). To this purpose, we use our laboratory frame waveguide dispersions β_j_(ω) to calculate the effective mass, scalar on-site potential, and magnetic vector potential around the reference waveguide and carrier frequency, as shown in the Supplementary Materials. We then diagonalize the Schrödinger equation (Eq. 6) for different values of ω′ to find the propagation constants in the comoving frame. The results for no coupling (*C* = 0) and for a finite coupling *C* are shown in Fig. 2 (D and E) for the same parameters as for Fig. 2 (B and C). The agreement between the two calculations is excellent, which further confirms the power of our configuration to generate a nontrivial synthetic magnetic field and thus realize a topological quantum Hall coupled wire model. As an aside, we mention that the engineering of the photonic band structure to have quantum Hall features is not unique to this model. In the Supplementary Materials, we present an analytically solvable toy model, whose band structure we also tune to resemble the coupled wire model (see Supplementary Materials). In the next section, we will investigate observable signatures of the synthetic magnetic field on optical quantities of experimental interest.

We note that the couplings used throughout this work are *C* ∼ 0.001 mm^−1^ in magnitude and require correspondingly long waveguides or state recycling techniques (*58*) to observe any dynamics, as discussed in the next section. While such a regime could be obtained in experiment by the use of sufficiently large interwaveguide spacings and long glass samples, the system might turn out to be sensitive to disorder in the optical medium, which we do not include in our model. If required, the analytical treatment in this section suggests several strategies to overcome the difficulty: The viable range of *C* and the required waveguide length are determined by the characteristic “kinetic energy” $\text{Δ}{\beta_{\mathrm{char}}}^{\prime }∼D_{j}\text{Δ}\omega^{\prime 2}$ in the temporal direction, which is determined by the bandwidth of the light source (here taken to be Δω′ ∼ 10^−3^ω_0_) and by the group velocity dispersion *D_j_* of the waveguides. The former can be increased using, e.g., shorter light pulses or wider tunable sources. The latter can be increased by using a different material with a stronger dispersion (that is, a lower Abbe number) than weakly dispersive fused silica glass or a narrower waveguide geometry with tighter transverse light confinement, e.g., on an integrated photonics platform (*59*). This would increase the characteristic kinetic energy $\text{Δ}{\beta_{\mathrm{char}}}^{\prime }$ and would correspondingly allow for larger values of the coupling *C* and shorter waveguide lengths.

### Observable signatures of the synthetic magnetic field

Having engineered our waveguide band structure in the comoving frame to resemble that of a quantum Hall coupled wire model, we now numerically demonstrate previously unseen optical effects that result from the synthetic magnetic field. These provide the smoking gun for nontrivial topological physics in this system.

### Cyclotron orbits

As a first example, we consider the optical equivalent of bulk cyclotron orbits that arise for a semiclassical charged particle in a magnetic field. As discussed above, equispaced Landau levels are present in the bulk of the coupled wire model (Fig. 1B). A wave packet prepared in a suitable superposition of Landau levels will then execute semiclassical cyclotron orbits, moving in a circular trajectory with the characteristic cyclotron frequency set by the level spacing and a (clockwise or anticlockwise) direction set by the sign of the effective magnetic field. In the presence of an additional synthetic electric field, the cyclotron motion will be supplemented by a so-called Hall drift, which is a sideways motion perpendicular to the direction of the applied electric field.

As we now show, such orbits naturally arise for photons in our system. To this purpose, we prepare an initial Gaussian wave packet in the *j* − ω′ space, spatially centered in the bulk of the array and with a central frequency located in the Landau level region of the bands. The Gaussian spatial width *s_j_* is taken of the order of the interwaveguide spacing, while the chosen frequency-space width *s*_ω′_ corresponds to a Gaussian pulse duration on the order of 100 fs. Such pulse durations are well within the range of standard techniques in ultrafast optics such as mode-locked lasers, and the light then has to be focused onto the input facet of the array with the appropriate spot waist to realize the desired Gaussian spatial profile. The wave packet is then evolved along ζ according to the Fourier-space comoving-frame evolution equation (Eq. 8) and Fourier-transformed into −τ space. The details of the numerical calculations throughout this section are discussed in Materials and Methods.

**Fig. 3.**
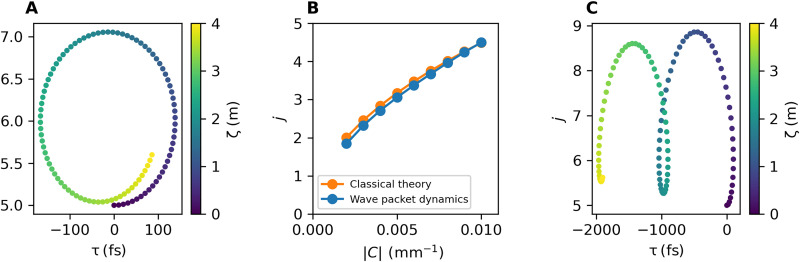
Bulk cyclotron orbits in the comoving frame. (**A**) In our wave packet dynamics with Eq. 9, we find cyclotron orbits, as shown here by the wave packet center of mass colored according to the position along the optical axis, ζ. (**B**) The frequencies of the cyclotron orbits extracted with a fit (blue) compared to the coupled wire model classical theory (orange), showing excellent agreement. (**C**) We apply a temperature gradient across the array, corresponding to an on-site potential. Tuning the strength of this potential introduces a Hall drift into the dynamics. The parameters used are the same as in Fig. 2. The example results in (A) and (C) use a coupling *C* = −0.002 mm^−1^. For the coupled-mode wave packet dynamics, the wave packet is prepared with an initial center of mass of *j*_0_ = 5 and ω′ = 0 and with widths of *s*_ω′_ = 1/500 × 10^15 ^Hz (corresponding to a Gaussian pulse length of 500 fs), and *s_j_* = 1. In (C), we use an electric potential of $\text{Δ}n_{j}\left(\frac{\omega_{0}}{c}\right)=0.001\;j\;{\mathrm{mm}}^{-1}$.

Figure 3A shows an example trajectory of the pulse center of mass, calculated for the waveguide array parameters used in Fig. 2. A clear cyclotron orbit is visible, where the amplitude of the oscillations along the spatial direction *j* is of the order of a waveguide, so they are detectable in experiments. To further characterize the oscillations, we repeat these simulations for different values of the interwaveguide coupling *C*, which physically corresponds to varying their spacing. For each calculation, the cyclotron frequency is extracted from a fit of the ζ evolution, the details of which are discussed in Materials and Methods. The blue line in Fig. 3B shows the value of the fitted cyclotron frequency as a function of the coupling *C*; as expected, it grows for increasing values of the coupling.

A deeper insight into the cyclotron oscillations can be obtained by comparing these numerical results with the prediction of an approximate classical calculation based on the equations of motion for a classical particle with constant, yet anisotropic masses and subject to the synthetic magnetic field according to the coupled wire model with no external potential (*60*). This calculation leads to the prediction

$$\omega_{c}=\frac{|B|}{\sqrt{m^{\left(\tau \right)}\;m^{\left(j\right)}}}=\;|B|\sqrt{2|C\;D_{j_{\mathrm{ref}}}|}$$ (14)

where ∣*m*^(τ),(*j*)^∣ = 1/∣*D*_*j*_ref__∣,1/(2∣*C*∣) are the absolute values of the effective masses in the temporal and spatial directions, respectively, and ℬ ≡ $\left[A_{j=N}^{\left(\tau \right)}-A_{j=1}^{\left(\tau \right)}\right]$/(*N* − 1) is the approximately uniform magnetic field corresponding to our magnetic vector potential. Depending on the relative sign of the masses in the two directions (namely, of *C* and *D*_*j*_ref__), the Landau levels appear for states displaying the same or opposite phases in neighboring wires. The result of this approximate calculation is shown as an orange line in Fig. 3B and displays a good agreement with the numerics for the full model (blue). The small deviation between the two curves is principally due to the minor differences between the ideal coupled-wire model bands and our full optical results in Fig. 2.

For the chosen system parameters, the typical period ζ*_c_* = 2π/ω*_c_* of the cyclotron oscillations is of the order of meters, which may be very demanding compared to the typical length of waveguide arrays fabricated in experiment. However, this difficulty could be mitigated by using state recycling techniques to increase the effective lengthscale explored in experiments (*58*) or by switching to different material platforms. The cyclotron orbit length scale is determined by the characteristic kinetic energy scale Δβ′_char_ which, as discussed above, may be increased using a wider operating bandwidth or samples with a stronger dispersion.

### Hall drift on the pulse arrival time

We now exploit another feature of quantum Hall physics to introduce a Hall drift into the cyclotron dynamics that we found above. As mentioned previously, if an additional electric field is applied to a particle in a quantum Hall system, we expect the particle to drift in the in-plane direction perpendicular to that field. We first consider applying a synthetic electric field across the array in the *j* direction, which will correspond to a drift in the τ direction. If this drift could be controlled, then natural applications of the resulting delay/advance include delay lines, which have widespread uses throughout optics, including interferometry, ultrafast optics, and telecommunications.

The Hall drift can be introduced, with controllable magnitude and direction, by imposing suitable perturbations to the waveguide array, e.g., a temperature gradient along *j*. This induces a corresponding spatial gradient of the refractive index Δ*n_j_* along *j*. Formally, this can be described by including an additional term of the form

$$i\frac{\partial {\tilde{a}}_{j}^{\prime }}{\partial \zeta }=\cdots +\text{Δ}n_{j}(\omega_{0}/c)\;{\tilde{a}}_{j}^{\prime }$$ (15)

to the right-hand side of our model equation (Eq. 8). An elementary calculation within the coupled wire model shows that the temporal drift under a synthetic electric field ℰ_therm_ = −(ω_0_/*c*)(*d*Δ*n_j_*/*dj*) in the spatial direction is equal to

$$\tau_{H}=-\zeta \;\frac{E_{\mathrm{therm}}}{B}$$ (16)

An example of this effect is illustrated in Fig. 3C, where we show the appearance of the Hall drift along τ under the effect of a potential gradient as small as Δ*n_j_*(ω_0_/*c*) = 0.001 *j* mm^−1^, corresponding to a refractive index perturbation of ∼10^−7^. We note that the upper limit on the perturbation strength is due to the size of the bandgap in Fig. 2C; a perturbation of the order of or larger than the gap will introduce nonadiabatic effects and blur out the effect shown in Fig. 3C.

### Hall drift in real space

In the previous subsection, we showed how the displacement along the τ direction can be introduced. Now, we propose a method to exploit the same quantum Hall effect to control the spatial displacement of a wave packet across the array, i.e., along the *j* direction, in response to a perturbation along the temporal τ direction.

**Fig. 4.**
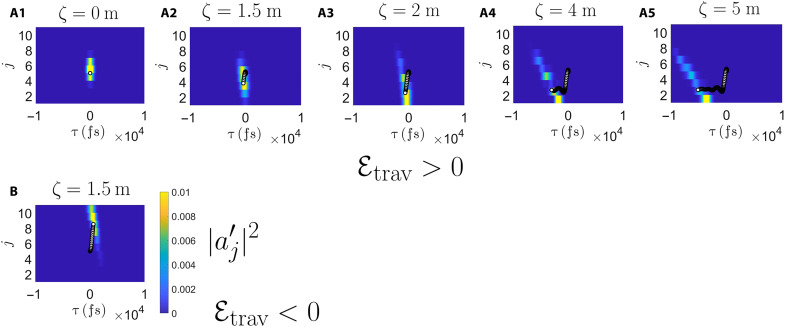
Controllable Hall drift of a wave packet in the comoving frame. (**A1 to A5**) We apply a traveling refractive index modulation to create an effective electric field in the τ direction, which causes a displacement of the wave packet across the waveguides as we expect for a quantum Hall system (see movie S1). We use the same system parameters as in Fig. 3, with an additional electric field ℰ_trav_ = 0.00001(fs mm)^−1^. (**B**) Reversing the sign of the refractive index modulation reverses the electric field to ℰ_trav_ = −0.00001(fs mm)^−1^ and, thus, the direction of displacement.

The idea of the scheme is to implement a spatial displacement in a reconfigurable way by means of a traveling refractive index perturbation. This could be realized in experiments by means of the electro-optic or acousto-optical effects, as was recently investigated in (*49*, *51*). In the simplest case, we consider a refractive index perturbation that is uniform across the array and travels along the waveguides at the same speed as the reference group velocity $v_{g}^{\left(\mathrm{ref}\right)}$

$$\text{Δ}n(z,t)=\text{Δ}\bar{n}\frac{z-v_{g}^{\left(\mathrm{ref}\right)}t}{\ell }$$ (17)

where 𝓁 is the length of the device in *z* and $\text{Δ}\bar{n}$ is the strength of the perturbation. Such a perturbation can be included in our model by adding a term of the form

$$i\frac{\partial a_{j}^{\prime }}{\partial \zeta }=\cdots -E_{\mathrm{trav}}\tau a_{j}^{\prime }$$ (18)

to the right-hand side of the Schrödinger equation (Eq. 6), where $E_{\mathrm{trav}}=\text{Δ}\bar{n}\;\omega_{0}\;v_{g}^{\left(\mathrm{ref}\right)}/(\ell c)$ is the synthetic electric field along the τ direction. Two examples of light propagation under such a perturbation are shown in Fig. 4 (A1, A2, and B) for the same magnitudes of the synthetic electric field but opposite signs. We see the wave packet, prepared in the system bulk, transported across the array toward larger or smaller *j*. These numerics are carried out using the frequency space Eq. 9, where the perturbation appears as a term of the form $iE_{\mathrm{trav}}\partial {\tilde{a}}_{j}^{\prime }/\partial \omega^{\prime }$ added to the right-hand side. Note also that the displacement appears despite the modulation being independent of *j*; this further confirms its origin from the synthetic magnetic field via the quantum Hall effect.

As an alternative scheme, the same effect could be realized by means of a variation of the magnetic vector potential $A_{j}^{\left(\tau \right)}$ in the Schrödinger equation (Eq. 6) during the evolution. According to the *t-z* mapping, this requires us to vary the waveguide properties along the waveguide axis. Analogously to classical electrodynamics, this produces an effective synthetic electric field ℰ_z−mod_ = −$dA_{j}^{\left(\tau \right)}$(ζ)/*d*ζ oriented along the τ direction, which, by the quantum Hall effect, induces a drift along the orthogonal spatial direction *j*. As an example, the spatial gradient of $A_{j}^{\left(\tau \right)}(z)$ in the *z* direction could be obtained in our setup by designing the waveguide parameters as

$$\delta n_{j}(z)=\delta n_{j-wz}(z=0)$$ (19)

$$\sigma_{j}(z)=\sigma_{j-wz}(z=0)$$ (20)

where evaluation of the waveguide parameters at the continuous-valued *j* − *wz* is obtained by interpolating their values at *z* = 0 in-between neighboring waveguides. Within the coupled wire model, this leads to

$$E_{z-\mathrm{mod}}=-\frac{dA_{j}^{\left(\tau \right)}(\zeta )}{d\zeta }=-wB$$ (21)

and the corresponding Hall drift in the spatial direction can be straightforwardly evaluated to be

$$j_{H}=-\zeta \frac{E_{z-\mathrm{mod}}}{B}$$ (22)

Despite their different optical implementation, it is worth noting that these two approaches are actually the same from the point of view of the band structure. Both of them are based on an adiabatic transport of the state along the band, corresponding to a change in the spatial position along the *j* direction, as indicated by the coloring of the bands in Fig. 2C.

### Propagation along the edge

The plots in Fig. 4 (A1, A2, and B) refer to relatively short propagation distances so that the Hall-drifted wave packets are still within the bulk of the waveguide array. At longer propagation distances, the wave packet can reach the spatial edge of the waveguide array at *j* = 1 or *j* = *N*. At this point, as is usual in topological systems under a synthetic electric field (*61*), the wave packet gets converted into an edge excitation that propagates along the edge. The ensuing fast chiral motion along the spatial edge of the system is clearly visible in the plots in Fig. 4 (A3 to A5), as well as in movie S1; because we are dealing with a spatial edge, the chiral motion is along the temporal τ direction, with a different sign depending on which edge the wave packet hits. Last, we note that we do not have an edge in the τ direction, so any advance or delay that we see from either the temperature gradient or from this chiral edge mode propagation could be of arbitrary size.

## DISCUSSION

In this work, we have demonstrated a novel framework to generate a synthetic magnetic field for light in a 1D array of coupled waveguides. On the basis of the *t-z* mapping of paraxial propagation of time-dependent optical pulses, a 2D model is obtained in the *j-t* plane spanned by the (discrete) waveguide index and the (continuous) temporal variable. With a suitable gradient of the waveguide properties across the array, an effective synthetic magnetic field is induced, which provides an optical realization of the quantum Hall coupled wire model. Observable signatures of the synthetic magnetic effects are anticipated as a chiral cyclotron motion in the *j-t* plane, a Hall drift in the temporal or spatial direction under the effect of a synthetic electric field directed along the array or in the temporal direction, and a fast propagation in chiral edge states.

From an experimental point of view, important advantages of our proposal over previous work on synthetic dimensions in photonics (*45*, *20*) can be pointed out. Building atop available schemes for topological photonics in waveguide arrays (*6*, *20*), our proposal only requires working with time-dependent pulses rather than monochromatic light, and in particular, it does not require any external dynamical modulation of the system and does not involve all the complexities of Floquet systems. Eventually, it will open new avenues for the spatiotemporal manipulation of optical pulses (*52*, *53*).

Generalization of our proposal to physically 2D waveguide arrays suggests a natural way to realize 3D models: Future work will be devoted to the investigation of topological models involving two discrete *j*_1,2_ coordinates and a continuous *t* one in our platform and the identification of observable optical signatures of the geometrical and topological properties of the peculiar features of 3D band structures such as Weyl points and Fermi arcs.

While this work was focused on a specific implementation of our proposed concept in laser-written waveguide array operating in the visible light domain, future work will be devoted to the identification of alternative realizations in different material systems and frequency domains, e.g., integrated photonics devices for infrared/visible light (*59*) or microwave waveguides (*62*), which may provide a more pronounced dispersion and strong nonlinearities.

In the long run, our proposal holds great promise in view of realizing exotic states of topological photonic matter. In contrast to topological models exploiting the light frequency as a synthetic dimension (*46*–*49*) where nonlinearities would typically result in long-range interactions along the frequency direction (*60*), the fact that the spatial coordinates of the topological model are encoded in the waveguide index *j* and the temporal variable *t* translates a spatially local nonlinearity of the medium into local interactions in the topological model. This feature is of extreme importance (*57*) when one is to scale up the interaction strength and realize strongly correlated states like fractional quantum Hall liquids of light (*63*, *64*).

## MATERIALS AND METHODS

### Selection of parameters for the model of a laser-written waveguide array

**Fig. 5.**
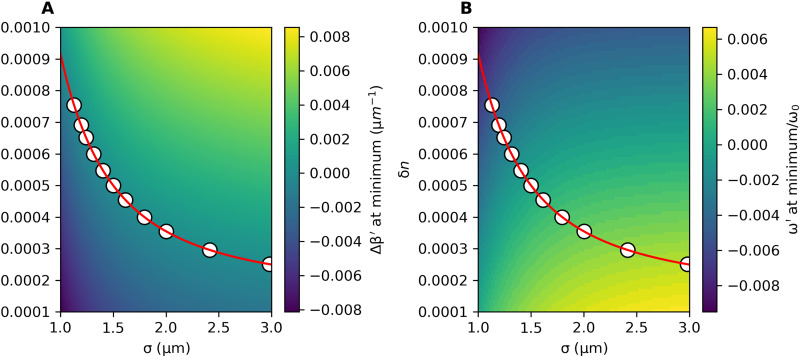
Selection of parameters for the model. (**A**) The comoving frame propagation constant at the minimum of the waveguide dispersion and (**B**) the corresponding ω′ value, each as a function of the two main model parameters. We choose the minimum Δβ′ = 0 contour (red) and choose points along it (white) that correspond to a uniform target spacing of Δω′ = 0.001 ω_0_ ω′. The reference waveguide is located in the center and has refractive index depth and width δ*n*_ref_ = 0.0005 and σ_ref_ = 1.5 μm, respectively. As discussed in detail in the text, material parameters are inspired from laser-written waveguides in fused silica glass, and a carrier frequency corresponding to a wavelength of 0.5 μm is considered.

To select parameters for the model defined in Eq. 12, we sweep out the (σ, δ*n*) parameter space, and for each point in the space, we find the dispersion β(ω) as described in Results. We choose the comoving frame to be defined by the reference waveguide with parameters δ*n*_ref_ = 0.0005 and σ_ref_ = 1.5 μm and choose a carrier frequency ω_0_ corresponding to a wavelength of 0.5 μm. Within this comoving frame, we identify for each value of the model parameters (σ, δ*n*) the minimum of the dispersion. We then plot the values of the Δβ′ propagation constant and of the ω′ frequency at this minimum as a function of (σ, δ*n*). The results of this are shown in Fig. 5.

To select the parameters for our array, we choose the zero contour of the minimum propagation constant surface to force all the curves to have their minima at the same value, corresponding to a vanishing on-site potential. We then sample *N* points from the chosen contour for an array of *N* waveguides, placing the reference waveguide in the center (the contour is shown in red, with the sampled points in white). We choose the points to enforce a chosen frequency spacing, Δω′, between adjacent dispersions (i.e., a chosen magnetic field strength). The end results in the laboratory frame are plotted in Fig. 2A and in the comoving frame in Fig. 2B.

### Details of wave packet dynamics simulations

**Fig. 6.**
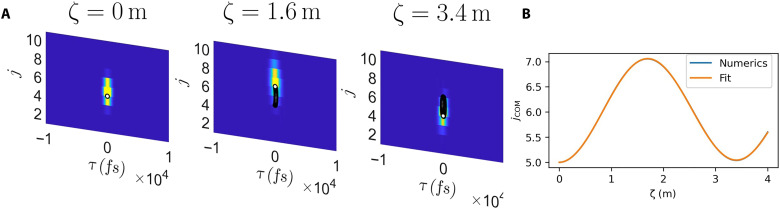
Details of the cyclotron orbit numerical simulations. (**A**) Examples of wave packet density plotted at different evolution times ζ starting from a Gaussian wave packet prepared in the bulk. We see part of a cyclotron orbit with a Hall drift. (**B**) Plot of the center of mass of the wave packet in (A) in the discrete direction as a function of ζ together with a fit to Eq. 26, showing that the fitting function captures our data very well and can be used to extract the cyclotron frequency. We use the same parameters as the wave packet dynamics simulations in Fig. 3, with a coupling *C* = −0.002 mm^−1^.

As discussed in Results, we use wave packet dynamics simulations to investigate bulk cyclotron orbits and other physical observables. We prepare a Gaussian wave packet of the form

$${\tilde{a}}_{j}^{\prime }(\zeta =0,\omega^{\prime })=Ae^{ik_{j}(j-j_{0})}e^{-\frac{{\left(j-j_{0}\right)}^{2}}{2s_{j}^{2}}}e^{ik_{\omega^{\prime }}(\omega^{\prime }-\omega_{0}^{\prime })}e^{-\frac{{\left(\omega^{\prime }-\omega_{0}^{\prime }\right)}^{2}}{2s_{\omega }^{2}\prime }}$$ (23)

where *A* is a normalization constant, *k*_(*j*,ω′)_ is the wave packet momentum in the two directions, (*j*, ω′)_0_ is the initial center-of-mass position, and *s*_(*j*,ω′)_ is the wave packet width along the two directions. We choose the wave packet to be localized in the *j* bulk and to have an initial frequency in the bulk part of the bands to target Landau level states. We then numerically propagate the wave packet through the array by discretizing the ω′ dimension into *M* ≫ 1 points and hence representing our initial wave packet as an *NM*-component column vector $\underline{a}(\zeta =0)$. We then evolve the initial vector via

$$\underline{a}(\zeta +\delta \zeta )=e^{-iH\delta \zeta }\underline{a}(\zeta )$$ (24)

where δζ is our small “time step” and *H* is the *NM* × *NM* matrix representing the right-hand side of the coupled mode equation (Eq. 8) in our finite difference basis. More precisely, we have

$$H=H_{\mathrm{diag}}-\left(\begin{array}{ccccc} 0 & C & 0 & \cdots & 0\\ C & \ddots & \ddots & \ddots & \vdots \\ 0 & \ddots & & & 0\\ \vdots & \ddots & & & C\\ 0 & \cdots & 0 & C & 0 \end{array}\right)⊗I_{\omega^{\prime }}$$ (25)

where *H*_diag_ is a diagonal matrix formed by placing (−1 times) the discretized comoving frame propagation constants β*_j_*(ω) − β_*j*_ref__(ω_0_) − ω′/$v_{g}^{\left(\mathrm{ref}\right)}$ along the main diagonal. The second term, representing the coupling between neighboring waveguides, is an *N* × *N* tridiagonal matrix with the couplings inserted on to the two diagonals either side of the main diagonal. We then Fourier-transform the state with respect to ω′ to map it into τ space and consider the corresponding *j* − τ density as a function of ζ. An example of the wave packet density for the optical model is shown in Fig. 6A, corresponding to the center-of-mass trajectory plotted in Fig. 3A. We use this density to calculate the wave packet center of mass as a function of ζ, *j*_COM_(ζ), and τ_COM_(ζ).

When investigating bulk cyclotron orbits, we fit to the *j*_COM_(ζ) trajectory using the function

$$f(\zeta )=A\sin(\omega_{c}\zeta +\phi )e^{-g\zeta }+B$$ (26)

and extract the cyclotron frequency ω*_c_*, an example of which is shown in Fig. 6B. We choose this function because we expect cyclotron orbits to be circular trajectories in (*j*, τ). We include an exponential damping factor to take into account the small damping seen in some of our numerics. We see that the fit captures our numerical data well, and it performs similarly well on all our numerics.

As discussed in Results, besides the bulk cyclotron orbits, we also investigate two other physical observables. The first is a traveling refractive index perturbation. We model this with a term of the form $iE_{\mathrm{trav}}\partial {\tilde{a}}_{j}^{\prime }/\partial \omega^{\prime }$ which corresponds, in the Schrödinger equation, to an effective electric field along the τ direction, −ℰ_trav_τ$a_{j}^{\prime }$. We include this term within our numerical scheme by representing the ∂/∂ω′ operator by a standard *M* × *M* finite difference first derivative matrix *d*_ω′_. We hence include the term in our total finite difference matrix as *H* → *H* + *iℰ*_trav_*I_j_* ⊗ *d*_ω′_, where *I_j_* is the *N* × *N* identity matrix. Last, we also consider applying a temperature gradient across the array, which we model with a term of the form $\text{Δ}n_{j}(\omega_{0}/c){\tilde{a}}_{j}^{\prime }$, where we choose Δ*n_j_* = *Uj*. We include this in our numerical scheme by *H* → *H* + *UJ* ⊗ *I*_ω′_, where *J* = diag (1, …, *N*) and *I*_ω′_ is the *M* × *M* identity matrix.

## Supplementary Materials

### This PDF file includes:

Supplementary Text Figs. S1 and S2 Legend for movie S1 References
