# Supplementary material for: Artificial gauge fields in the t-z mapping for optical pulses: Spatiotemporal wave packet control and quantum Hall physics
Source: Sci Adv. 2023 Oct 20;9(42):eadj0360. doi: 10.1126/sciadv.adj0360 (PMC10588944; doi:10.1126/sciadv.adj0360)
Supplement: Supplementary file 1 — Supplementary Text Figs. S1 and S2 Legend for movie S1 References [file sciadv.adj0360_sm.pdf]

Science Advances
AAAS

Supplementary Materials for

Artificial gauge fields in the *t-z* mapping for optical pulses: Spatiotemporal wave packet control and quantum Hall physics

Christopher Oliver *et al.*

Corresponding author: Christopher Oliver, chroli.phys@gmail.com

Sci. Adv. **9**, eadj0360 (2023)
DOI: 10.1126/sciadv.adj0360

The PDF file includes:

Supplementary Text
Figs. S1 and S2
Legend for movie S1
References

Other Supplementary Material for this manuscript includes the following:

Movies S1

We cover two topics in this Supplemental Material. In Sec. 1, we discuss a simple, analytically-solvable toy model for a 1D coupled waveguide array, which we use to demonstrate that the idea of engineering the co-moving frame band structure to have quantum Hall features is quite gen-eral and not specific to the particular model chosen. For the other section, we return to the experimentally-motivated model introduced in the Main Text. In Sec. 2, we show the calcula-tion of the Schrödinger equation effective mass, magnetic vector potential and on-site potential which we use in some of the results in the Main Text.

1 An Analytical Toy Model for Coupled Waveguides

To further demonstrate the engineering of non-trivial magnetic field effects from tuning waveguide parameters as discussed in the Main Text, we now consider a simple, analytical model for a coupled waveguide array, consisting of metal waveguides each embedding a medium of frequency-independent refractive index n_j. We can exactly solve the paraxial Helmholtz equation to find that the TE_{10} modes, which we choose for simplicity, have the dispersion *(65)*:

$$\beta_j(\omega) = \frac{n_j}{c}\sqrt{\omega^2 - \omega_c^{(j)2}}, \tag{S1}$$

where $\omega_c^{(j)} = \pi c/(L_x^{(j)} n_j)$ is the cutoff frequency in waveguide j, below which no modes can propagate. The cutoff frequency depends on the waveguide width $L_x^{(j)}$ which we allow one to spatially vary in order to engineer a non-trivial magnetic field.

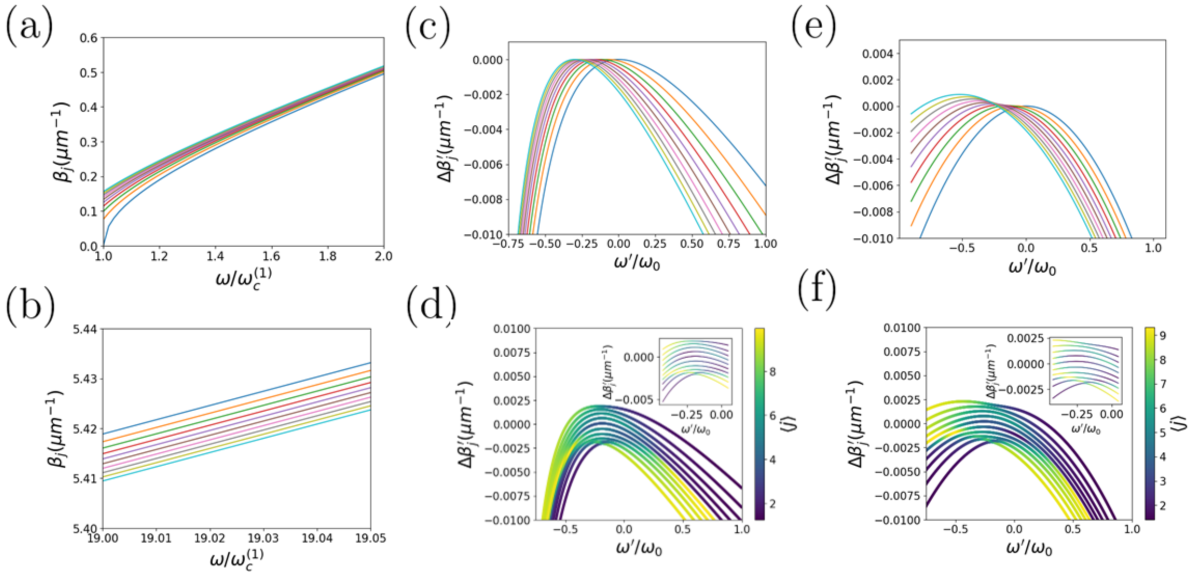

Figure S1: **Propagation constants for the analytical toy model in both reference frames** (a): Propagation constants in the lab frame for our toy model. The blue labelled curve is the dispersion for waveguide $j = 1$. (b): The dispersion curves in (a) for larger frequencies, showing that the order of the curves has reversed and hence that the dispersions cross over each other. (c): Direct mapping of the dispersions in (a) to the co-moving frame using our modified Doppler shift. We see the maxima of the dispersion curves all have the same $\Delta\beta_j'$ value as we expect. (d): Results in (c) now including a coupling between neighbouring waveguides, showing avoided crossings. This results in a band structure resembling the coupled wire model, including bulk Landau levels (c.f. inset) and chiral edge modes in the gap. (e): Propagation constants in the co-moving frame for this model calculated using our Schödinger equation without any coupling, showing qualitative agreement with our analytical results in (c). (f): Results in (e) including a coupling between neighbouring waveguides. Throughout, we use $N = 10$ waveguides with $L_0 = 10\mu$m, $\Delta L = 1\mu$m and $n_1 = 1.5$, and we take $\omega_0 = 10\omega_c^{(1)}$. We take the coupling to be $C = 10^3\mu\text{m}^{-1}$.

We can now transform the dispersion into the co-moving frame using the mapping in the Main Text (Eq. 10). Note that, in the co-moving frame, we actually plot $\Delta\beta_j'(\omega') = \beta_j'(\omega') - \beta_{\text{ref}}'(0)$ for consistency with our definition of propagation constants in our Schrödinger equation. In our calculations here, we choose the reference waveguide to be the $j = 1$ waveguide in the array and we take $\omega_0 = 10\,\omega_c^{(1)}$ as the carrier frequency.

We can then use the transformed dispersions to tune the refractive index n_j such that the

maxima of the dispersions in the co-moving frame all take the same value, which makes the resultant band structure as similar as possible to the coupled wire model (discussed in the Main Text). We tune our refractive index profile as:

$$n_j = \frac{n_1}{\sqrt{1 + \left(\frac{\omega_c^{(1)}}{\omega_0}\right)^2 \left(\left(\frac{L_x^{(j)}}{L_x^{(1)}}\right)^2 - 1\right)}} \tag{S2}$$

which we calculate by differentiating our transformed propagation constants with respect to ω' and enforcing that the maxima are all equal in $\Delta\beta'_j$. We also choose the waveguide widths $L_x^{(j)} = L_0 + \Delta L\sqrt{j}$ to ensure that the spacing between adjacent dispersion curves is approximately constant, to approach the case of a uniform magnetic field. The resulting dispersions in both frames are shown in Fig. S1(a), (b) and (c). In the lab frame, we see a set of dispersion curves that cross over each other (c.f. the reversed order of the curves in (a) vs. (b)). In the co-moving frame, we have a set of curves with maxima that are all at the same $\Delta\beta'_j$ value as expected from our chosen refractive index profile (c.f. panel (c)). Introducing a coupling between neighbouring waveguides results in avoided crossings (Fig. S1(d)), and we see a band structure that resembles that of the quantum Hall coupled wire model (c.f. Fig. 1(b) in the Main Text). In particular, in the inset of the figure, we see flat Landau level states in the bulk and chiral edge states in the gap, which is characteristic of quantum Hall systems. Furthermore, panel (a) suggests that a stronger dispersion could be obtained by working at a lower value of $\omega_0/\omega_c^{(1)}$, which corresponds to a tighter waveguide confinement, that is, a smaller L_0.

We can also calculate the band structure from our Schrödinger equation (Eq. 6 in the Main Text) by using our propagation constants (Eq. S1) to calculate m_j, $A_j^{(\tau)}$ and V_j, and then diagonalising the resulting Hamiltonian. Example results are shown in Fig S1(e) and (f), without and with an inter-waveguide coupling respectively. Comparing to the corresponding exact results in (c) and (d), we see that, as expected, the Schrödinger equation captures the dispersions very well close to $\omega' = 0$ because the truncated Taylor expansion is most accurate there. Moving

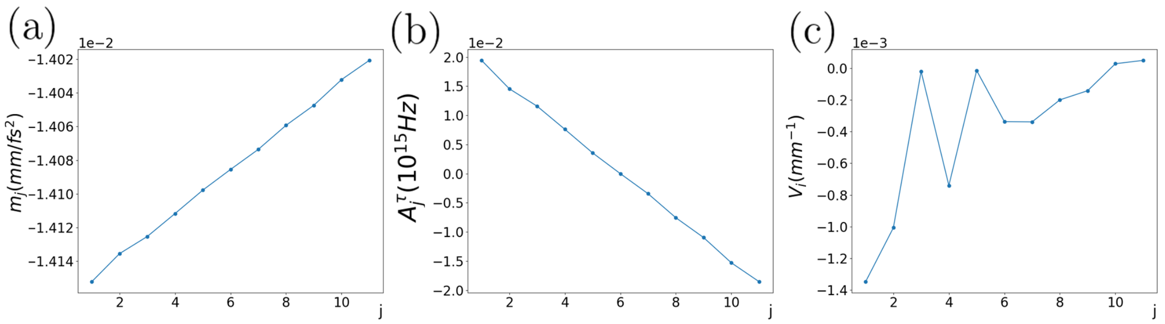

Figure S2: **Schrödinger equation quantities for the model in the Main Text** Effective mass, magnetic vector potential and on-site potential in panels (a), (b) and (c) respectively for our Schrödinger equation, calculated from data in Fig. 2 in the Main Text. We see a weakly-varying effective mass, near-linear magnetic vector potential (corresponding to a uniform magnetic field) and a small residual on-site potential.

away from $\omega' = 0$ in either direction leads to disagreement between the two approaches, most notably in the heights of the maxima not being identical in (e), leading to dispersion even in the flat Landau level states in (f). Finally, we note that the bands we find for this model are inverted relative to the bands for the experimentally-motivated model we consider in the Main Text (i.e. the bands for the toy model have stationary points that are maxima, not minima). This is because the group velocity dispersions in the two models have opposite signs. Overall, these results therefore demonstrate that we do not require a complex model to engineer the kind of physics we find here.

2 Schrödinger equation effective mass, magnetic vector potential and on-site potential

In this section, we show the results of using the waveguide dispersions calculated in the Main Text to find the effective mass, magnetic vector potential and on-site potential in the Schrödinger equation, which we then use to calculate some of the results in Fig. 2. We use the lab-frame propagation constants (Fig. 2(a)) and evaluate the three quantities using Eq. 7 in the Main

Text. The results are shown in Fig. S2. We see a near-constant effective mass (with variation on the order 1% across the array), a magnetic vector potential that is very close to linear in j (corresponding to a uniform magnetic field), and a very small on-site potential.

Caption for Movie S1: Controllable Hall drift of a wavepacket in the co-moving frame. We apply a travelling refractive index modulation to create an effective electric field in the τ direction, which causes a displacement of the wavepacket across the waveguides as we expect for a quantum Hall system. We use the same system parameters as Fig. 3 in the Main Text, with an additional electric field $\mathcal{E}_{\text{trav}} = 0.00001(\text{fs mm})^{-1}$.

REFERENCES AND NOTES

1. R. W. Boyd, *Nonlinear Optics* (Academic Press, ed. 3, 2008)

2. A. Szameit, S. Nolte, Discrete optics in femtosecond-laser-written photonic structures. *J. Phys. B: At. Mol. Opt. Phys.* **43**, 163001 (2010).

3. T. Schwartz, G. Bartal, S. Fishman, M. Segev, Transport and Anderson localization in disordered two-dimensional photonic lattices. *Nature* **446**, 52–55 (2007).

4. L. Levi, M. Rechtsman, B. Freedman, T. Schwartz, O. Manela, M. Segev, Disorder-enhanced transport in photonic quasicrystals. *Science* **332**, 1541–1544 (2011).

5. M. Verbin, O. Zilberberg, Y. E. Kraus, Y. Lahini, Y. Silberberg, Observation of topological phase transitions in photonic quasicrystals. *Phys. Rev. Lett.* **110**, 076403 (2013).

6. M. C. Rechtsman, J. M. Zeuner, A. Tünnermann, S. Nolte, M. Segev, A. Szameit, Strain-induced pseudomagnetic field and photonic Landau levels in dielectric structures. *Nat. Photonics* **7**, 153–158 (2013).

7. S. Mukherjee, M. Di Liberto, P. Öhberg, R. R. Thomson, N. Goldman, Experimental observation of Aharonov-Bohm cages in photonic lattices. *Phys. Rev. Lett.* **121**, 075502 (2018).

8. A. Szameit, M. C. Rechtsman, O. Bahat-Treidel, M. Segev, PT-symmetry in honeycomb photonic lattices. *Phys. Rev. A* **84**, 021806 (2011).

9. M. Segev, B. Crosignani, A. Yariv, B. Fischer, Spatial solitons in photorefractive media. *Phys. Rev. Lett.* **68**, 923–926 (1992).

10. H. S. Eisenberg, Y. Silberberg, R. Morandotti, A. R. Boyd, J. S. Aitchison, Discrete spatial optical solitons in waveguide arrays. *Phys. Rev. Lett.* **81**, 3383–3386 (1998).

11. N. K. Efremidis, S. Sears, D. N. Christodoulides, J. W. Fleischer, M. Segev, Discrete solitons in photorefractive optically induced photonic lattices. *Phys. Rev. E.* **66**, 046602 (2002).

12. D. Christodoulides, F. Lederer, Y. Silberberg, Discretizing light behaviour in linear and nonlinear waveguide lattices. *Nature* **424**, 817–823 (2003).

13. J. W. Fleischer, M. Segev, N. K. Efremidis, D. N. Christodoulides, Observation of two-dimensional discrete solitons in optically induced nonlinear photonic lattices. *Nature* **422**, 147–150 (2003).

14. Y. Lahini, A. Avidan, F. Pozzi, M. Sorel, R. Morandotti, D. N. Christodoulides, Y. Silberberg, Anderson localization and nonlinearity in one-dimensional disordered photonic lattices. *Phys. Rev. Lett.* **100**, 013906 (2008).

15. B. Freedman, G. Bartal, M. Segev, R. Lifshitz, D. N. Christodoulides, J. W. Fleischer, Wave and defect dynamics in nonlinear photonic quasicrystals. *Nature* **440**, 1166–1169 (2006).

16. P.-E. Larré, I. Carusotto, Optomechanical signature of a frictionless flow of superfluid light. *Phys. Rev. A* **91**, 053809 (2015).

17. Q. Fontaine, P.-É. Larré, G. Lerario, T. Bienaimé, S. Pigeon, D. Faccio, I. Carusotto, É. Giacobino, A. Bramati, Q. Glorieux, Interferences between Bogoliubov excitations in superfluids of light. *Phys. Rev. Res.* **2**, 043297 (2020).

18. M. C. Braidotti, R. Prizia, C. Maitland, F. Marino, A. Prain, I. Starshynov, N. Westerberg, E. M. Wright, D. Faccio, Measurement of Penrose superradiance in a photon superfluid. *Phys. Rev. Lett.* **128**, 013901 (2022).

19. J. Steinhauer, M. Abuzarli, T. Aladjidi, T. Bienaimé, C. Piekarski, W. Liu, E. Giacobino, A. Bramati, Q. Glorieux, Analogue cosmological particle creation in an ultracold quantum fluid of light. *Nat. Commun.* **13**, 2890 (2022).

20. T. Ozawa, H. M. Price, A. Amo, N. Goldman, M. Hafezi, L. Lu, M. C. Rechtsman, D. Schuster, J. Simon, O. Zilberberg, I. Carusotto, Topological photonics. *Rev. Mod. Phys.* **91**, 015006 (2019).

21. H. Price, Y. Chong, A. Khanikaev, H. Schomerus, L. J. Maczewsky, M. Kremer, M. Heinrich, A. Szameit, O. Zilberberg, Y. Yang, B. Zhang, A. Alù, R. Thomale, I. Carusotto, P. St-Jean, A. Amo, A.

Dutt, L. Yuan, S. Fan, X. Yin, C. Peng, T. Ozawa, A. Blanco-Redondo, Roadmap on topological photonics. *J. Phys. Photonics* **4**, 032501 (2022).

22. M. C. Rechtsman, J. M. Zeuner, Y. Plotnik, Y. Lumer, D. Podolsky, F. Dreisow, S. Nolte, M. Segev, A. Szameit, Photonic Floquet topological insulators. *Nature* **496**, 196–200 (2013).

23. N. H. Lindner, G. Refael, V. Galitski, Floquet topological insulator in semiconductor quantum wells. *Nat. Phys.* **7**, 490–495 (2011).

24. Y. Lumer, Y. Plotnik, M. C. Rechtsman, M. Segev, Self-localized states in photonic topological insulators. *Phys. Rev. Lett.* **111**, 243905 (2013).

25. S. Mukherjee, M. C. Rechtsman, Observation of unidirectional solitonlike edge states in nonlinear Floquet topological insulators. *Phys. Rev. X* **11**, 041057 (2021).

26. S. Mukherjee, M. C. Rechtsman, Observation of Floquet solitons in a topological bandgap. *Science* **368**, 856–859 (2020).

27. J. Noh, S. Huang, D. Leykam, Y. D. Chong, K. P. Chen, M. C. Rechtsman, Experimental observation of optical Weyl points and Fermi arc-like surface states. *Nat.Phys.* **13**, 611–617 (2017).

28. E. Lustig, L. J. Maczewsky, J. Beck, T. Biesenthal, M. Heinrich, Z. Yang, Y. Plotnik, A. Szameit, M. Segev, Three-dimensional photonic topological insulator induced by lattice dislocations. arXiv:2204.13762 [physics.optics] (28 April 2022).

29. Z. Yang, E. Lustig, Y. Lumer, M. Segev, Photonic Floquet topological insulators in a fractal lattice. *Light Sci. Appl.* **9** 128 (2020).

30. Z. Fu, N. Fu, H. Zhang, Z. Wang, D. Zhao, S. Ke, Extended SSH model in non-Hermitian waveguides with alternating real and imaginary couplings. *Appl. Sci.* **10**, 3425 (2020).

31. J. M. Zeuner, M. C. Rechtsman, Y. Plotnik, Y. Lumer, S. Nolte, M. S. Rudner, M. Segev, A. Szameit, Observation of a topological transition in the bulk of a non-Hermitian system. *Phys. Rev. Lett.* **115**, 040402 (2015).

32. S. Weidemann, M. Kremer, T. Helbig, T. Hofmann, A. Stegmaier, M. Greiter, R. Thomale, A. Szameit, Topological funneling of light. *Science* **368**, 311–314 (2020).

33. E. J. Meier, F. A. An, A. Dauphin, M. Maffei, P. Massignan, T. L. Hughes, B. Gadway, Observation of the topological Anderson insulator in disordered atomic wires. *Science* **362**, 929–933 (2018).

34. S. Stützer, Y. Plotnik, Y. Lumer, P. Titum, N. H. Lindner, M. Segev, M. C. Rechtsman, A. Szameit, Photonic topological Anderson insulators. *Nature* **560**, 461–465 (2018).

35. Y. E. Kraus, Y. Lahini, Z. Ringel, M. Verbin, O. Zilberberg, Topological states and adiabatic pumping in quasicrystals. *Phys. Rev. Lett.* **109**, 106402 (2012).

36. O. Zilberberg, S. Huang, J. Guglielmon, M. Wang, K. P. Chen, Y. E. Kraus, M. C. Rechtsman, Photonic topological boundary pumping as a probe of 4D quantum Hall physics. *Nature* **553**, 59–62 (2018).

37. M. Wimmer, H. M. Price, I. Carusotto, U. Peschel, Experimental measurement of the Berry curvature from anomalous transport. *Nat.Phys.* **13**, 545–550 (2017).

38. T. Kitagawa, M. A. Broome, A. Fedrizzi, M. S. Rudner, E. Berg, I. Kassal, A. Aspuru-Guzik, E. Demler, A. G. White, Observation of topologically protected bound states in photonic quantum walks. *Nat. Commun.* **3**, 882 (2012).

39. Y. Lai, H. A. Haus, Quantum theory of solitons in optical fibers. I. Time-dependent Hartree approximation. *Phys. Rev. A* **40**, 844–853 (1989).

40. Y. Lai, H. A. Haus, Quantum theory of solitons in optical fibers. II. Exact solution. *Phys. Rev. A* **40**, 854–866 (1989).

41. P.-E. Larré, I. Carusotto, Propagation of a quantum fluid of light in a cavityless nonlinear optical medium: General theory and response to quantum quenches. *Phys. Rev. A* **92**, 043802 (2015).

42. C. L. Kane, R. Mukhopadhyay, T. C. Lubensky, Fractional quantum Hall effect in an array of quantum wires. *Phys. Rev. Lett.* **88**, 036401 (2002).

43. J. C. Y. Teo, C. L. Kane, From Luttinger liquid to non-Abelian quantum Hall states. *Phys. Rev. B* **89**, 085101 (2014).

44. J. C. Budich, A. Elben, M. Łącki, A. Sterdyniak, M. A. Baranov, P. Zoller, Coupled atomic wires in a synthetic magnetic field. *Phys. Rev. A* **95**, 043632 (2017).

45. L. Yuan, Q. Lin, M. Xiao, S. Fan, Synthetic dimension in photonics. *Optica* **5**, 1396 (2018).

46. T. Ozawa, H. M. Price, N. Goldman, O. Zilberberg, I. Carusotto, Synthetic dimensions in integrated photonics: From optical isolation to four-dimensional quantum hall physics. *Phys. Rev. A* **93**, 043827 (2016).

47. L. Yuan, Y. Shi, S. Fan, Photonic gauge potential in a system with a synthetic frequency dimension. *Opt. Lett.* **41**, 741–866 744 (2016).

48. A. Dutt, L. Yuan, K. Y. Yang, K. Wang, S. Buddhiraju, J. Vučković, S. Fan, Creating boundaries along a synthetic frequency dimension. *Nat. Commun.* **13**, 3377 (2022).

49. K. Wang, B. A. Bell, A. S. Solntsev, D. N. Neshev, B. J. Eggleton, A. A. Sukhorukov, Multidimensional synthetic chiral-tube lattices via nonlinear frequency conversion. *Light Sci. Appl.* **9**, 132 (2020).

50. Y. Hu, C. Reimer, A. Shams-Ansari, M. Zhang, M. Loncar, Realization of high-dimensional frequency crystals in electro-optic microcombs. *Optica* **7**, 1189–1194 (2020).

51. F. S. Piccioli, A. Szameit, I. Carusotto, Topologically protected frequency control of broadband signals in dynamically modulated waveguide arrays. *Phys. Rev. A* **105**, 053519 (2022).

52. T. Ozawa, Artificial magnetic field for synthetic quantum matter without dynamical modulation. *Phys. Rev. A* **103**, 033318 (2021).

53. L. Nemirovsky, M.-I. Cohen, Y. Lumer, E. Lustig, M. Segev, Synthetic-space photonic topological insulators utilizing dynamically invariant structure. *Phys. Rev. Lett.* **127**, 093901 (2021).

54. D. R. Hofstadter, Energy levels and wave functions of Bloch electrons in rational and irrational magnetic fields. *Phys. Rev. B* **14**, 2239–2249 (1976).

55. T. Chalopin, T. Satoor, A. Evrard, V. Makhalov, J. Dalibard, R. Lopes, S. Nascimbene, Probing chiral edge dynamics and bulk topology of a synthetic hall system. *Nat. Phys.* **16**, 1017–1021 (2020).

56. M. Polyanskiy, *Optical Constants of Fused Silica* (*Fused Quartz*), https://refractiveindex.info/?shelf=glass book=fused_silica page=Malitson [accessed 20 April 2023].

57. T. Ozawa, H. M. Price, Topological quantum matter in synthetic dimensions. *Nat. Rev. Phys.* **1**, 349–357 (2019).

58. S. Mukherjee, H. Chandrasekharan, P. Öhberg, N. Goldman, R. Thomson, State-recycling and time-resolved imaging in topological photonic lattices. *Nat. Commun.* **9**, 4209 (2018).

59. E. Pelucchi, G. Fagas, I. Aharonovich, D. Englund, E. Figueroa, Q. Gong, H. Hannes, J. Liu, C.-Y. Lu, N. Matsuda, J.-W. Pan, F. Schreck, F. Sciarrino, C. Silberhorn, J. Wang, K. D. Jöns, The potential and global outlook of integrated photonics for quantum technologies *Nat. Rev. Phys.* **4**, 194–208 (2022).

60. T. Ozawa, I. Carusotto, Synthetic dimensions with magnetic fields and local interactions in photonic lattices. *Phys. Rev. Lett.* **118**, 013601 (2017).

61. D. De Bernardis, F. Piccioli, P. Rabl, I. Carusotto, Chiral quantum optics in the bulk a photonic quantum Hall system. arXiv p. 2302.14863 (24 July 2023).

62. M. Esposito, A. Ranadive, L. Planat, N. Roch, Perspective on traveling wave microwave parametric amplifiers. *Appl. Phys. Lett.* **119**, 120501 (2021).

63. I. Carusotto, C. Ciuti, Quantum fluids of light. *Rev. Mod. Phys.* **85**, 299–366 (2013).

64. I. Carusotto, A. A. Houck, A. J. Kollár, P. Roushan, D. I. Schuster, J. Simon, Photonic materials in circuit quantum electrodynamics. *Nat. Phys.* **16**, 268–279 (2020).

65. I. Grant, W. Phillips, Electromagnetism (Wiley, Chicester, ed. 2, 2004).
